# Prophylactic pegfilgrastim reduces febrile neutropenia in ramucirumab plus docetaxel after chemoimmunotherapy in advanced NSCLC: post hoc analysis from NEJ051

**DOI:** 10.1038/s41598-024-54166-x

**Published:** 2024-02-15

**Authors:** Keita Miura, Ou Yamaguchi, Keita Mori, Atsushi Nakamura, Motohiro Tamiya, Tomohiro Oba, Noriko Yanagitani, Hideaki Mizutani, Takashi Ninomiya, Tomosue Kajiwara, Kentaro Ito, Akihiko Miyanaga, Daisuke Arai, Hiroaki Kodama, Kunihiko Kobayashi, Kyoichi Kaira

**Affiliations:** 1 Department of Respiratory Medicine, Juntendo University Graduate School of Medicine, Tokyo, Japan; 2 Department of Respiratory Medicine, Comprehensive Cancer Center, Saitama Medical University International Medical Center, 1397-1, Yamane, Hidaka, 350-1298 Japan; 3 Clinical Research Center, Shizuoka Cancer Center, Nagaizumi, Japan; 4 Department of Pulmonary Medicine, Sendai Kousei Hospital, Sendai, Japan; 5 Department of Thoracic Oncology, Osaka International Cancer Institute, Osaka, Japan; 6 Department of Respiratory Medicine, Saitama Red Cross Hospital, Saitama, Japan; 7 Department of Thoracic Medical Oncology, The Cancer Institute Hospital of Japanese Foundation for Cancer Research, Tokyo, Japan; 8 Department of Thoracic Oncology, Saitama Cancer Center, Saitama, Japan; 9 Department of Thoracic Oncology and Medicine, National Hospital Organization Shikoku Cancer Center, Matsuyama, Japan; 10 Department of Internal Medicine, Niigata Cancer Center Hospital, Niigata, Japan; 11 Respiratory Center, Matsusaka Municipal Hospital, Matsusaka, Mie Japan; 12 Department of Pulmonary Medicine and Oncology, Graduate School of Medicine, Nippon Medical School, Tokyo, Japan; 13 Department of Internal Medicine, Saiseikai Utsunomiya Hospital, Utsunomiya, Japan; 14 Division of Thoracic Oncology, Shizuoka Cancer Center, Shizuoka, Japan

**Keywords:** Pegfilgrastim, Ramucirumab plus docetaxel, Neutropenia, Febrile neutropenia, Non-small cell lung cancer, Cancer, Oncology

## Abstract

Ramucirumab plus docetaxel (RD) can cause febrile neutropenia (FN), which frequently requires the prophylactic administration of pegfilgrastim. However, the effects of prophylactic pegfilgrastim on FN prevention, therapeutic efficacy, and prognosis after RD have not been fully evaluated in patients with advanced non-small-cell lung cancer (NSCLC). Two hundred and eighty-eight patients with advanced NSCLC who received RD as second-line therapy after platinum-based chemotherapy plus PD-1 blockade were included. Patients were divided into groups with and without prophylactic pegfilgrastim, and adverse events, efficacy, and prognosis were compared between both groups. Of the 288 patients, 247 received prophylactic pegfilgrastim and 41 did not. The frequency of grade 3/4 neutropenia was 62 patients (25.1%) in the pegfilgrastim group and 28 (68.3%) in the control group (*p* < 0.001). The frequency of FN was 25 patients (10.1%) in the pegfilgrastim group and 10 (24.4%) in the control group (*p* = 0.018). The objective response rate was 31.2% and 14.6% in the pegfilgrastim and control groups (*p* = 0.039), respectively. The disease control rate was 72.9% in the pegfilgrastim group and 51.2% in the control group (*p* = 0.009). Median progression free survival was 4.3 months in the pegfilgrastim group and 2.5 months in the control group (*p* = 0.002). The median overall survival was 12.8 and 8.1 months in the pegfilgrastim and control groups (*p* = 0.004), respectively. Prophylactic pegfilgrastim for RD reduced the frequency of grade 3/4 neutropenia and febrile neutropenia and did not appear to be detrimental to patient outcome RD.

Clinical Trial Registration Number: UMIN000042333.

## Introduction

Ramucirumab plus docetaxel (RD) therapy is an option for patients with previously treated advanced non-small cell lung cancer (NSCLC). A randomized phase 3 study (REVEL) demonstrated significantly better overall survival (OS) than with docetaxel alone in patients with previously treated NSCLC^1^. Real-world data (NEJ051, REACTIVE) of 288 RD-treated patients with NSCLC in second-line treatment after chemoimmunotherapy showed an objective response rate of 28.8% (95% confidence interval (CI):23.7–34.4)^2^. Therefore, RD is a confirmed as one of treatment options for patients with previously treated advanced NSCLC.

Besides, a phase 2 study (JVCG) of RD in Japan reported a high frequency of neutropenia and febrile neutropenia (FN)^3^. The frequency of all-grade neutropenia in the JVCG study was 94.7%^3^. The frequency of grade 3 or higher neutropenia and FN have previously been reported as 89.5% and 34.2%, respectively^3^. Although the docetaxel dose was 60 mg/m^2^ in Japan, hematological toxicity was observed more frequently than that in the REVEL study, requiring palliative management.

The American Society of Clinical Oncology (ASCO) guidelines recommend the prophylactic use of granulocyte colony stimulating factors (G-CSFs) when the risk of FN exceeds 20%^4^. Pegfilgrastim (PEG) is a long-acting preparation that has a prolonged blood elimination half-life owing to the chemical binding of polyethylene glycol to the N-terminus of granulocyte colony-stimulating factor (G-CSF)^5^. PEG is subcutaneously administered at a dose of 3.6 mg once on day 2 per chemotherapy cycle. One small prospective study reported that the incidence of FN with PEG in RD therapy was 5%^6^, and several retrospective studies have described the incidence of FN as 0–7.4%^7–11^. Although these exploratory studies identified the prevention of FN in RD therapy by the administration of PEG, the sample sizes of these previous investigations were limited; thus, it remains unclear whether the clinical usefulness of PEG in RD treatment could be confirmed as standard care. Aside from the preventive effect of FN after RD treatment, there is insufficient evidence to determine whether PEG could affect the efficacy and outcome of RD. A large sample size is needed to elucidate the clinical utility of PEG prophylaxis after RD initiation. Based on this background, we conducted a post-hoc analysis to evaluate the clinical significance of PEG prophylaxis after the initiation of RD in patients with previously treated NSCLC using a large sample size, as previously described^2^.

## Results

### Patient demographics

Table 1 shows the demographics of patients classified according to the presence or absence of PEG treatment. Two hundred and forty-seven patients (85.8%) received prophylactic PEG treatment, and 41 (14.2%) (control group) did not. The number of patients with a history of smoking was significantly higher in the control group than in the PEG group (*p* = 0.025). There were no significant differences in other factors. Of the 247 patients with PEG prophylaxis, 223 (90.3%) underwent prophylaxis during the first cycle of RD and 24 (9.7%) during the second cycle. Therefore 65 patients (41 control plus 24 s cycle) did not receive PEG prophylaxis during the first cycle of RD. Table 2 shows the characteristics of the patients who did/did not receive PEG prophylaxis during the first cycle. The proportion of patients aged ≥ 75 years was significantly higher in the PEG than in the control group (*p* = 0.013). The number of patients with a history of smoking was significantly higher in the control than in the PEG group (*p* = 0.043). There were no statistically significant differences in other factors.

**Table 1:** Comparison of patient characteristics with or without pegfilgrastim.

Variables		Total (n = 288)	PEG		
			Yes (n = 247)	No (n = 41)	*p*-value
Age	< 75yrs / > 75yrs	262/26	222 /25	40/1	0.145
Gender	Male/female	222/66	186/61	36/5	0.107
Performance status	0–1/ 2–3	269/19	230/17	39/2	> 0.999
Histology	AC/non-AC	199/89	172/75	27/14	0.715
Stage	III /IV/ope. rec	236/52	207/40	29/12	0.051
Smoking	Yes/no	237/51	198/49	39/2	**0.025**
T factor*	T1-2/T3-4	142/126	122/125	20/21	> 0.999
N factor*	N0/N1-3	63/225	54/193	9/32	> 0.999
M factor*	M0-1a-b/M1c	173/115	147/100	26/15	0.732
Any RT	Yes/no	81/207	64/183	17/24	0.059
TPS, PD-L1	> 50 / < 50/UN	60/204/24	50/174/23	10/30/1	0.313
Taxane in 1st line	Yes/no	94/194	84/163	10/31	0.281
Bevacizumab in 1st line	Yes/no	37/251	34/213	3/38	0.321
PD-1 blockade in 1st line	PD-1/PD-L1	236/52	200/47	36/5	0.383

yrs, years; PEG, pegfilgrastim; AC, adenocarcinoma; Ope rec., recurrence after operation; RT, radiation therapy; TPS, tumor proportion score; PD-L1, programmed death-ligand 1; UN, unknown; PD-L1, programmed death-ligand 1; bold character, statistical significance. *At the beginning of first-line therapy.

**Table 2:** Patient characteristics compared between with and without pegfilgrastim from the first cycle.

Variables		Total (n = 288)	PEG		
			Yes (n = 223)	No (n = 65)	*p*-value
Age	< 75 yrs / > 75 yrs	262/26	198/25	64/1	**0.013**
Gender	M/F	222/66	169/54	53/12	0.403
Performance status	0–1/ 2–3	269/19	206/17	63/2	0.262
Histology	AC/non-AC	199/89	157/66	42/23	0.446
Stage	III/IV/Ope rec	236/52	184/39	52/13	0.714
Smoking	Yes/no	237/51	178/45	59/6	**0.043**
T factor*	T1-2/ T3-4	142/126	111/112	31/34	0.780
N factor*	N0/N1-3	63/225	48/175	15/50	0.865
M factor*	M0-1a-b/M1c	173/115	134/89	39/26	> 0.999
Any RT	Yes/no	81/207	58/165	23/42	0.159
TPS, PD-L1	> 50/ < 50/UN	60/204/24	43/160/20	17/44/4	0.420
Taxane in 1st line	Yes/no	94/194	75/148	19/46	0.550
Bevacizumab in 1st line	Yes/no	37/251	32/191	5/60	0.207
PD-1 blockade in 1st line	PD-1/PD-L1	236/52	180/43	56/9	0.364

yrs, years; PEG, pegfilgrastim; AC, adenocarcinoma; Ope rec., recurrence after operation; RT, radiation therapy; TPS, tumor proportion score; PD-L1, programmed death-ligand 1; UN, unknown; PD-L1, programmed death-ligand 1; bold character, statistical significance. *At the beginning of first-line therapy.

### Implementation status of drug delivery

The starting dose of docetaxel for RD was 60 mg/m^2^ in 270 (93.8%) patients and < 60 mg/m^2^ in 18 (6.3%) patients. Of the 247 patients with PEG prophylaxis, the docetaxel dose was 60 mg/m^2^ in 233 (94.3%) and < 60 mg/m^2^ in 14 (5.7%). Of the 41 controls, the docetaxel dose was 60 mg/m^2^ in 37 (90.2%) and < 60 mg/m^2^ in 4 (9.8%) patients. The median number of RD cycles in all patients was 4 (range, 1–22). The median number of RD cycles with and without PEG prophylaxis was 5 (range, 1–22) compared with 2 (range, 1–19) for patients without PEG prophylaxis (*p* = 0.019). RD treatment was discontinued in 270/288 patients (93.8%); 93.9% (232/247 patients) in the PEG group and 92.7% (38/41 patients) in the control group. Sixty-three (27.2%) of 232 PEG-treated patients and 10 (26.3%) of the 38 control patients discontinued treatment. The majority of these patients discontinued due to progressive disease and AE.

### Therapeutic efficacy

Table 3 shows the efficacy of RD. The ORR and DCR for RD were 28.8% (95% CI 23.7–34.4) and 69.8% (95% CI 64.1–75.0), respectively. The ORR was 31.2% (95% CI 25.7–37.2) in the PEG group and 14.6% (95% CI 6.5–28.8) in the control group (*p* = 0.039). DCR was 72.9% (95% CI 67.0–78.0) in the PEG group and 51.2% (95% CI 36.5–65.7) in the control group (*p* = 0.009). Table S2 compares the efficacy in patients with and without PEG prophylaxis after the first cycle of RD. The ORR was 31.4% (95% CI 25.6–37.8) in the PEG group (n = 223) and 20.0% (95% CI 11.9–31.4) in the control group (n = 65) (*p* = 0.087). DCR was 72.2% (95% CI 66.0–77.7) in the PEG group and 61.5% (95% CI 49.4–72.4) in control group (*p* = 0.124).

**Table 3:** Efficacy of ramucirumab plus docetaxel.

Response	Total (n = 288)	PEG		
		Yes (n = 247)	No (n = 41)	*p*-value
CR	1	1	0	–
PR	82	76	6	–
SD	118	103	15	–
PD	73	55	18	–
NE	14	12	2	–
ORR, % (95% CI)	28.8 (23.9–34.3)	31.2 (25.7–37.2)	14.6 (6.5–28.8)	**0.039**
DCR, % (95% CI)	69.8 (64.3–74.8)	72.9 (67.0–78.0)	51.2 (36.5–65.7)	**0.009**

PEG, pegfilgrastim; CR, complete response; PR, partial response; SD, stable response; PD, progressive disease; NE, not evaluable; ORR, objective response rate; DCR, disease control rate; CI confidence interval. Significant values are in bold.

### Survival analysis

**Figure 1:**
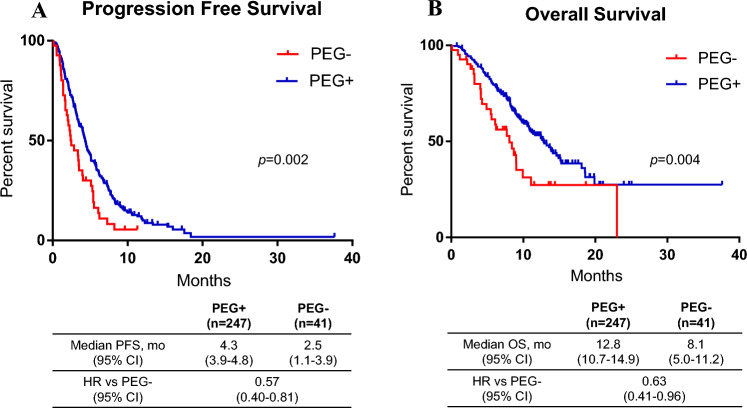
Kaplan–Meier curves showing progression-free survival (**A**) and overall survival (**B**) classified according to the presence or absence of prophylactic pegfilgrastim (PEG) in ramucirumab plus docetaxel treatment. Median progression free survival was 4.3 months (95% CI 3.9–4.8) in the PEG prophylaxis group and 2.5 months (95% CI 1.1–3.9) in the control group (*p* = 0.002). Median overall survival was 12.8 months (95% CI 10.7–14.9) in the PEG prophylaxis group and 8.1 months (95% CI 5.0–11.2) in the control group (*p* = 0.004).

Figure 1 shows Kaplan–Meier curves for PFS and OS classified according to whether prophylactic PEG was administered or not. Median PFS was 4.3 months (95% CI 3.9–4.8) in the PEG group and 2.5 months (95% CI 1.1–3.9) in the control group (significant difference (HR 0.57; 95% CI 0.40–0.81; *p* = 0.002)). Median OS was 12.8 months (95% CI 10.7–14.9) in the PEG group and 8.1 months (95% CI 5.0–11.2) in the control group [significant difference (HR 0.63; 95% CI 0.41–0.96; *p* = 0.004)]. Figure S1 shows the Kaplan–Meier survival curves with and without PEG administration after the first cycle. Median PFS was 4.4 months (95% CI 3.9–4.8) in the PEG group and 3.4 months (95% CI 2.7–4.0) in the control group (significant difference (HR 0.77; 95% CI 0.58–1.03; *p* = 0.022)). Median OS was 13.8 months (95% CI 11.8–15.8) in the PEG treatment group and 8.7 months (95% CI 7.2–10.1) in the control group (significant difference (HR 0.63; 95% CI 0.44–0.90; *p* = 0.001)). Figures S2 and S3 show the Kaplan–Meier survival curves according to PEG administration in the adenocarcinoma (AC) and non-adenocarcinoma (non-AC) groups. Figures S4 and S5 show the Kaplan–Meier survival curves based on PEG administration after the first cycle among the different histology. The PFS was not significantly different between the PEG prophylaxis and control groups in AC patients, but a statistically significant difference was observed between groups in PFS and OS for non-AC patients and OS for AC patients.

### Relationship between first-line treatment and RD

The PFS (175 days vs. 167 days, *p* = 0.095) and ORR (55.1% vs. 43.9%, *p* = 0.236) to first-line treatments were not significantly different between the patients who used and did not use PEG. Moreover, the response of PR or CR to first-line treatments was observed in 54 (70.1%) of 77 patients and 4 (66.7%) of 6 patients with CR or PR to RD in the PEG group and control group, respectively, without statistical significance.

### Adverse events

Table 4 shows hematological toxicities. The frequencies of grade 3/4 leukopenia, anemia, thrombocytopenia, neutropenia, and FN in all patients were 23.6%, 6.3%, 2.1%, 31.3%, and 12.2%, respectively. The frequencies of grade 3/4 leukopenia were 21.5% in the PEG prophylaxis group and 36.6% in the control group (*p* = 0.046), those of grade 3/4 neutropenia were 25.1% in the PEG group and 68.3% in the control group (*p* < 0.001), and those of FN were 10.1% in the PEG prophylaxis group and 24.4% in the control group (*p* = 0.018). No statistically significant differences were observed in the non-hematological AE between the PEG and control groups (Table 5).

**Table 4:** Hematological toxicities of ramucirumab plus docetaxel.

Incidence of Hematological toxicities and FN	Total n = 288 (%)	PEG		
		Yes n = 247 (%)	No n = 41(%)	*p*-value
Any grade				
Leukopenia	122 (42.4)	91 (36.8)	31 (75.6)	** < 0.001**
Anemia	147 (51.0)	125 (50.6)	22 (53.7)	0.740
Thrombocytopenia	94 (32.6)	87 (35.2)	7 (17.1)	**0.030**
Neutropenia	119 (41.3)	86 (34.8)	33 (80.5)	** < 0.001**
FN	35 (12.2)	25 (10.1)	10 (24.4)	**0.018**
Grade 3/4				
Leukopenia	68 (23.6)	53 (21.5)	15 (36.6)	**0.046**
Anemia	18 (6.3)	17 (6.9)	1 (2.4)	0.485
Thrombocytopenia	6 (2.1)	6 (2.4)	0 (0.0)	0.599
Neutropenia	90 (31.3)	62 (25.1)	28 (68.3)	** < 0.001**
FN	35 (12.2)	25 (10.1)	10 (24.4)	**0.018**

PEG, pegfilgrastim; FN, febrile neutropenia; bold character, statistical significance.

**Table 5:** Non-hematological toxicities of ramucirumab plus docetaxel.

Incidence of non-hematological toxicities	Total n = 288 (%)	PEG		
		Yes n = 247 (%)	No n = 41 (%)	*p*-value
Anorexia	125 (43.4)	110 (44.5)	15 (36.6)	0.397
Fatigue	115 (39.9)	100 (40.5)	15 (36.6)	0.732
Alopecia	107 (37.2)	94 (38.1)	13 (31.7)	0.489
AST increased	86 (29.9)	74 (30.0)	12 (29.3)	> 0.999
Mucositis oral	84 (29.2)	71 (28.7)	13 (31.7)	0.713
Edema limbs	67 (23.3)	62 (25.1)	5 (12.2)	0.075
Nausea	63 (21.9)	55 (22.3)	8 (19.5)	0.839
ALT increased	60 (20.8)	54 (21.9)	6 (14.6)	0.406
Diarrhea	54 (18.8)	44 (17.8)	10 (24.4)	0.386
Constipation	46 (16.0)	41 (16.6)	5 (12.2)	0.646
Rash	42 (14.6)	39 (15.8)	3 (7.3)	0.230
Dysphasia	34 (11.8)	31 (12.6)	3 (7.3)	0.440
Pneumonitis	31 (10.8)	25 (10.1)	6 (14.6)	0.413
Vomiting	20 (6.9)	19 (7.7)	1 (2.4)	0.327
Arthralgia	18 (6.3)	16 (6.5)	2 (4.9)	> 0.999
Myalgia	11 (3.8)	11 (4.5)	0 (0.0)	0.374
Peripheral neuropathy	11 (3.8)	11 (4.5)	0 (0.0)	0.374
Blood bilirubin increased	5 (1.7)	5 (2.0)	0 (0.0)	> 0.999
Edema face	3 (1.0)	3 (1.2)	0 (0.0)	> 0.999
Paronychia	3 (1.0)	3 (1.2)	0 (0.0)	> 0.999
Proteinuria	61 (21.2)	54 (21.9)	7 (17.1)	0.544
Epistaxis	49 (17.0)	45 (18.2)	4 (9.8)	0.261
Hypertension	31 (10.8)	25 (10.1)	6 (14.6)	0.413
Creatinine increased	24 (8.3)	22 (8.9)	2 (4.9)	0.548
Bronchial hemorrhage	18 (6.3)	17 (6.9)	1 (2.4)	0.485
Gastrointestinal hemorrhage	8 (2.8)	7 (2.8)	1 (2.4)	> 0.999
Thromboembolic event	3 (1.0)	3 (1.2)	0 (0.0)	> 0.999
Heart failure	1 (0.3)	1 (0.4)	0 (0.0)	> 0.999

PEG, pegfilgrastim; AST, aspartate aminotransferase; ALT, alanine aminotransferase.

## Discussion

This post-hoc analysis identified the clinical usefulness of prophylactic PEG in patients with advanced NSCLC who received RD immediately after PD-1 blockade plus platinum-based chemotherapy. We found that the frequency of FN was 10.1% in the PEG prophylaxis group and 24.4% in the control group. The incidence of FN in the REVEL and JVCG studies was 16% and 34.2%, respectively^1,3^. The incidence of FN in patients who underwent prophylactic PEG in our study was lower than that reported in previous prospective studies^1,3^. Although there were no significant differences in the patient characteristics between the two groups, prophylactic PEG potentially improved compliance with RD administration by preventing the occurrence of FN. Our large-scale study confirmed that prophylactic PEG can improve the therapeutic efficacy and minimize the toxicity of RD treatment after first line chemoimmunotherapy. However, we guess that the different incidence of FN between JVCG and REVEL study may be caused by the ethnic different between Caucasian and Asian patients. Therefore, we should recommend the use of prophylactic PEG only to Asian patients.

Several studies have described the palliative usefulness of RD treatment for FN prevention^5,7–11^. When prophylactic PEG was administered for RD treatment, the incidence of FN was < 10%, with an average of approximately 5%^5,7–11^. Without prophylactic PEG, the incidence of FN was approximately 25%^7,8,10,11^. Considering the results of previous and current studies, prophylactic PEG immediately after RD treatment should be considered routinely necessary for the prevention of FN and the continuous administration of RD. A retrospective study has reported that the prophylactic use of PEG reduced the hospitalization rate for multiple cancer types^12^. In our study, the number of dosing cycles of RD tended to be higher in the PEG group, although the frequency of discontinuation due to AE or progressive disease was similar in the PEG prophylaxis and PEG control groups. The optimal timing for chemotherapeutic administration of prophylactic PEG may affect the efficacy and prognosis of RD treatment. Mouri et al. compared the efficacy and outcome of RD between 29 patients with PEG prophylaxis and 4 patients without^8^. Because of the very small size of the control group, the ORR, PFS, and OS were not significantly different between the two groups. Moreover, Sakaguchi et al*.* reported that 95 of their 114 (83.3%) patients received prophylactic PEG, whereas 19 (16.7%) did not^9^. Although their study did not include information regarding the incidence of FN in the control group, the use of prophylactic PEG was significantly associated with favorable PFS and OS^9^. However, there are several concerns about previous studies. The number and regimens of prior treatments before RD initiation differed in each study, and there was no uniformity in prior immune checkpoint inhibitor (ICI) treatment. A recent study suggested the potential for increased efficacy of RD by prior ICI treatment^13^. Considering the regimens used in previous studies^8,9^, prior immunotherapy may affect the therapeutic efficacy of RD treatment, regardless of the administration of prophylactic PEG. In our study, the regimen administered just before RD was unified as PD-1 blockade plus platinum-based chemotherapy. Interestingly, we found that prophylactic PEG affected the therapeutic efficacy of RD immediately after chemoimmunotherapy. A recent preclinical study demonstrated that PEG enhances the antitumor activity of immunotherapy with antibody-dependent cellular cytotoxicity (ADCC) or phagocytosis (ADCP)^14^. As a possible mechanism, stimulation with PEG was related to significant enhancement of leukocytes in the spleen and the mobilization of activated monocytes or granulocytes from the spleen to the tumor bed^14^. Although it remains unknown whether PEG could potentiate the antitumor activity of PD-1 blockade, the synergistic relationship between PEG and prior immunotherapy is an interesting topic requiring further investigation to elucidate a possible mechanism. Currently, immunotherapy or chemoimmunotherapy are the standard first-line treatments for the care of patients with advanced NSCLC. Unlike previous studies, our study included all patients receiving first-line chemoimmunotherapy. Therefore, our population size meets the requirements for actual clinical practice and will be helpful to physicians.

A new device for convenient PEG administration has been developed. An on-body injector (OBI) is a device worn by patients after chemotherapy that automatically administers PEG approximately 27 h later^15^. OBI was not inferior to conventional manual injection in terms of pharmacokinetic safety^15^. Prospective studies in breast cancer, prostate cancer, lung cancer, and non-Hodgkin’s lymphoma have reported that the OBI reduces neutropenia compared with conventional FN strategies^16^. The use of the OBI has been suggested to improve adherence and compliance. Thus, preventive effects of the OBI on FN are expected in patients with cancer receiving strong myelosuppressive chemotherapy.

This study has limitations. First, the number of patients in the PEG (n = 247) and control groups (n = 41) was unbalanced because of a retrospective sub-analysis. Second, we could not determine the reasons for choosing PEG, as the decision to use prophylactic PEG depended on the physicians at each institution. An imbalance in the patients in each group may have affected the results of our study. This difference between the PEG prophylaxis and control groups may be associated with differences in efficacy and outcomes of RD treatment. Finally, we could not collect information on whether patients had undergone filgrastim treatment at neutropenia or FN onset in the control group. Future prospective comparative studies are required to verify the therapeutic efficacy and outcomes of PEG prophylaxis in RD treatment. This study is a retrospective assessment, and the major difference between the patients who received and did not receive PEG depended on the judgment of chief physicians at different institutions. No precious definition of PEG administration remains unclear, thus, it may bias the results of our study.

Prophylactic PEG for RD therapy significantly reduced the frequency of neutropenia and FN in patients with advanced NSCLC after chemotherapy. The use of prophylactic PEG does not appear to be detrimental to patient outcome of RD in such patients. Prophylactic PEG is clinically recommended for the prevention of FN after RD.

## Methods

### Design

A total of 62 Japanese institutions participated in this post-hoc analysis (OnlineAppendix Table S1). We identified 288 patients who received RD as second-line therapy after first-line platinum-based chemotherapy plus PD-1 blockade therapy between January 2017 and August 2020. Our analysis was performed using the same sample as that used in a previously described study^2^. The following were administered as first-line treatments:

- Pembrolizumab plus cisplatin or carboplatin plus pemetrexed therapy (KEYNOTE-189)^17^;
- Pembrolizumab plus carboplatin plus nab-paclitaxel or paclitaxel therapy (KEYNOTE-407)^18^;
- Atezolizumab plus carboplatin plus paclitaxel plus bevacizumab therapy (IMpower150)^19^;
- Atezolizumab plus carboplatin plus nab-paclitaxel therapy (IMpower130)^20^; or
- Atezolizumab plus carboplatin plus pemetrexed therapy (IMpower132)^21^.

Patients who received PEG prophylaxis after RD initiation were compared with those who did not. Clinical data up to March 31, 2021, were extracted from the medical records. This study was approved by the institutional ethics committees of Saitama Medical University International Medical Center. The requirement for informed consent was waived by the ethics committee of the Saitama Medical University International Medical Center owing to the retrospective nature of the study. We would like to confirm that all procedures and methodologies used in this study were carried out in strict compliance with the relevant guidelines and regulations as stipulated by the journal’s editorial policy. We have obtained all required permissions and have ensured that our methods are transparent, ethical, and rigorous.

### Treatment and evaluation

All patients received combined chemotherapy with anti-PD-1/PD-L1 antibodies as first-line treatment. The KEYNOTE-189^17^, KEYNOTE-407^18^, IMpower150^19^, IMpower130^20^, and IMpower132 regimens^21^ were intravenously administered. RD (ramucirumab 10 mg/kg and docetaxel 60 mg/m^2^) was intravenously administered as second-line treatment. PEG (3.6 mg, G-LASTA™, Kyowa Kirin Co. Ltd., Tokyo, Japan), a prophylactic granulocyte colony-stimulating factor used after RD initiation, was administered based on the judgement of the chief doctor at the individual institution, and its subcutaneous administration was performed per chemotherapy cycle in all patients.

Physical examinations, complete blood counts, biochemical tests, and the recording of adverse events (AE) were performed at the discretion of the chief physicians at the respective institutions. Toxicity was graded based on the Common Terminology Criteria for Adverse Events, version 4.0. The tumor response was examined according to the Response Evaluation Criteria in Solid Tumors version 1.1.^22^.

### Prognostic endpoints’ definition

The periods considered for overall survival (OS) versus progression free survival (PFS) events were as follows: initiation of RD till the date of mortality due to any cause versus initiation of RD till the dates of exacerbation or mortality due to any cause, or initiation of third-line treatment.

### Statistics

To summarize the patient background and treatment results, we calculated the number of patients in each category and the median and maximum values in the continuous data. We further calculated the objective response rate (ORR) and disease control rate (DCR); [%, 95% CI]. The Kaplan–Meier method was used to obtain the survival curves. The significance levels of the tests and confidence coefficients for interval estimation were 5% and 95%, respectively, on both sides. Fisher’s exact test was used to examine the association between categorical variables. GraphPad Prism (version 7.0; GraphPad Software, San Diego, CA, USA) and JMP 14.0 (SAS Institute Inc., Cary, North Carolina, USA) were used for statistical analyses.

### Supplementary Information

Supplementary Figures.

Supplementary Tables.

Supplementary Information 3.

## Data Availability

The datasets used and/or analysed during the current study available from the corresponding author on reasonable request.
