# Supplementary material for: Prophylactic pegfilgrastim reduces febrile neutropenia in ramucirumab plus docetaxel after chemoimmunotherapy in advanced NSCLC: post hoc analysis from NEJ051
Source: Sci Rep. 2024 Feb 15;14:3816. doi: 10.1038/s41598-024-54166-x (PMC10869351; doi:10.1038/s41598-024-54166-x)
Supplement: Supplementary file 1 — Supplementary Figures. [file 41598_2024_54166_MOESM1_ESM.pptx]

## Slide 1
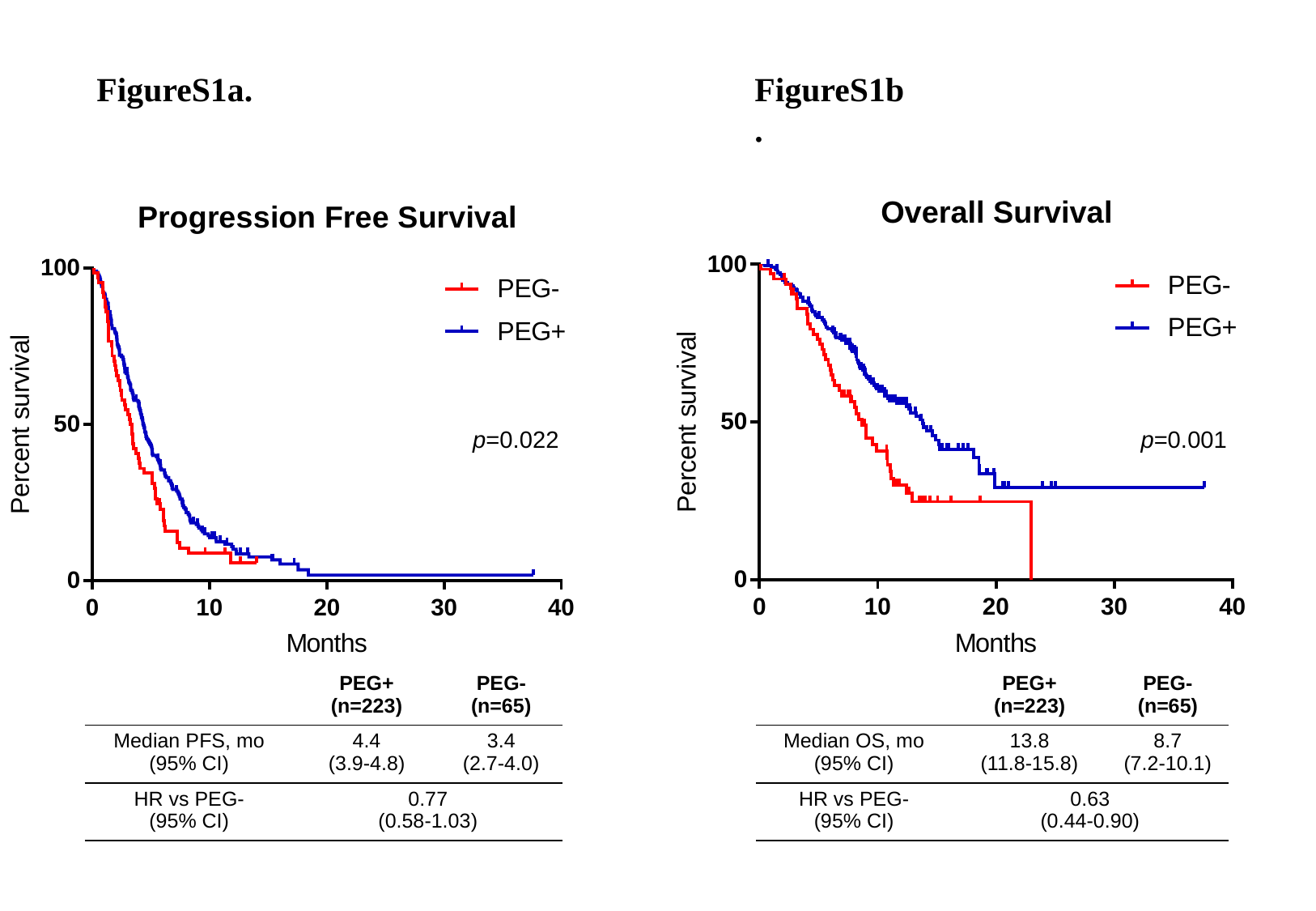

FigureS1a.
FigureS1b.
p=0.001
p=0.022
| | PEG+ (n=223) | PEG- (n=65) |
| --- | --- | --- |
| Median PFS, mo (95% CI) | 4.4 (3.9-4.8) | 3.4 (2.7-4.0) |
| HR vs PEG- (95% CI) | 0.77 (0.58-1.03) | |
| | PEG+ (n=223) | PEG- (n=65) |
| --- | --- | --- |
| Median OS, mo (95% CI) | 13.8 (11.8-15.8) | 8.7 (7.2-10.1) |
| HR vs PEG- (95% CI) | 0.63 (0.44-0.90) | |

## Slide 2
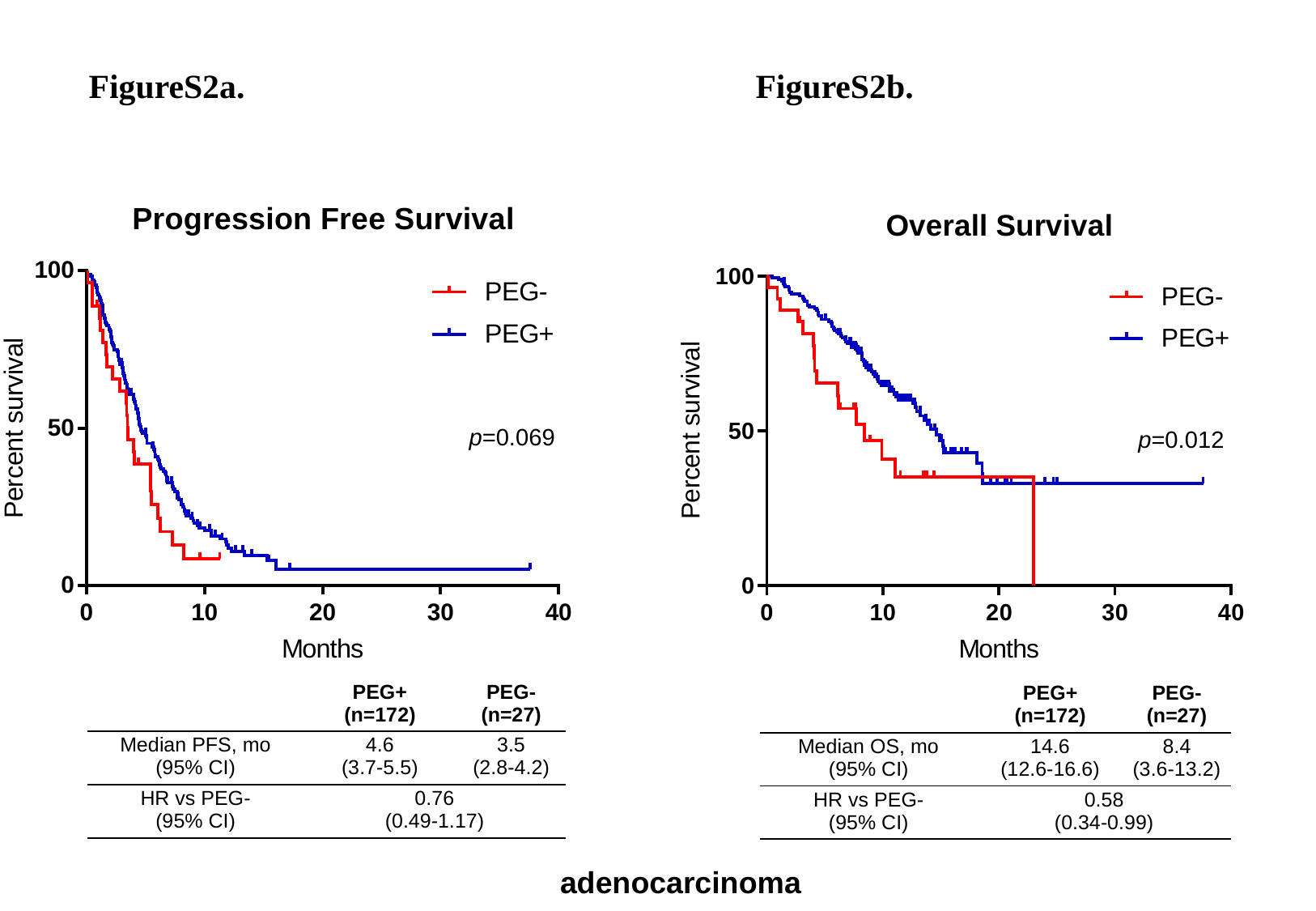

FigureS2a.
FigureS2b.
p=0.069
p=0.012
| | PEG+ (n=172) | PEG- (n=27) |
| --- | --- | --- |
| Median PFS, mo (95% CI) | 4.6 (3.7-5.5) | 3.5 (2.8-4.2) |
| HR vs PEG- (95% CI) | 0.76 (0.49-1.17) | |
| | PEG+ (n=172) | PEG- (n=27) |
| --- | --- | --- |
| Median OS, mo (95% CI) | 14.6 (12.6-16.6) | 8.4 (3.6-13.2) |
| HR vs PEG- (95% CI) | 0.58 (0.34-0.99) | |
adenocarcinoma

## Slide 3
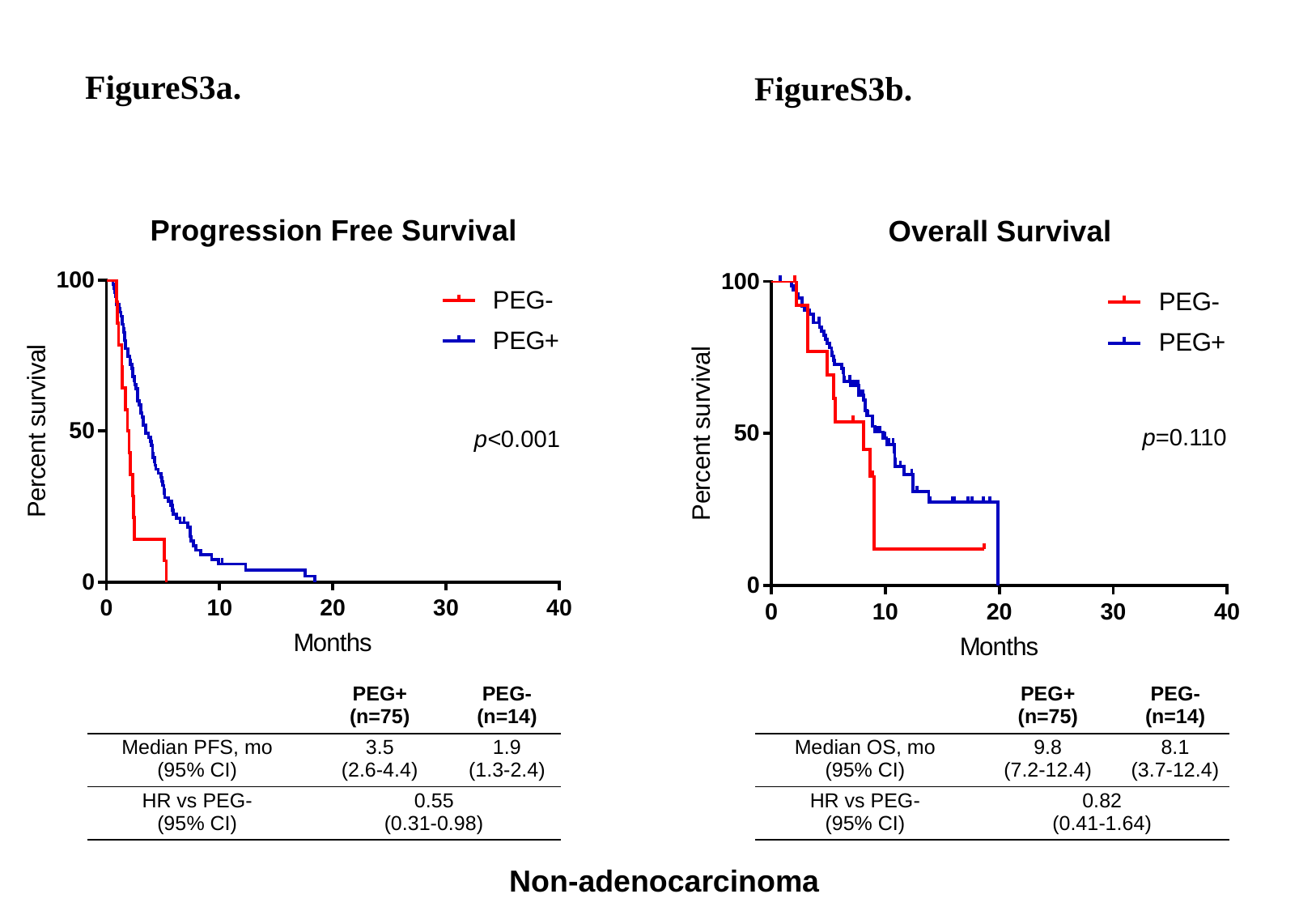

FigureS3a.
FigureS3b.
p=0.110
p<0.001
| | PEG+ (n=75) | PEG- (n=14) |
| --- | --- | --- |
| Median PFS, mo (95% CI) | 3.5 (2.6-4.4) | 1.9 (1.3-2.4) |
| HR vs PEG- (95% CI) | 0.55 (0.31-0.98) | |
| | PEG+ (n=75) | PEG- (n=14) |
| --- | --- | --- |
| Median OS, mo (95% CI) | 9.8 (7.2-12.4) | 8.1 (3.7-12.4) |
| HR vs PEG- (95% CI) | 0.82 (0.41-1.64) | |
Non-adenocarcinoma

## Slide 4
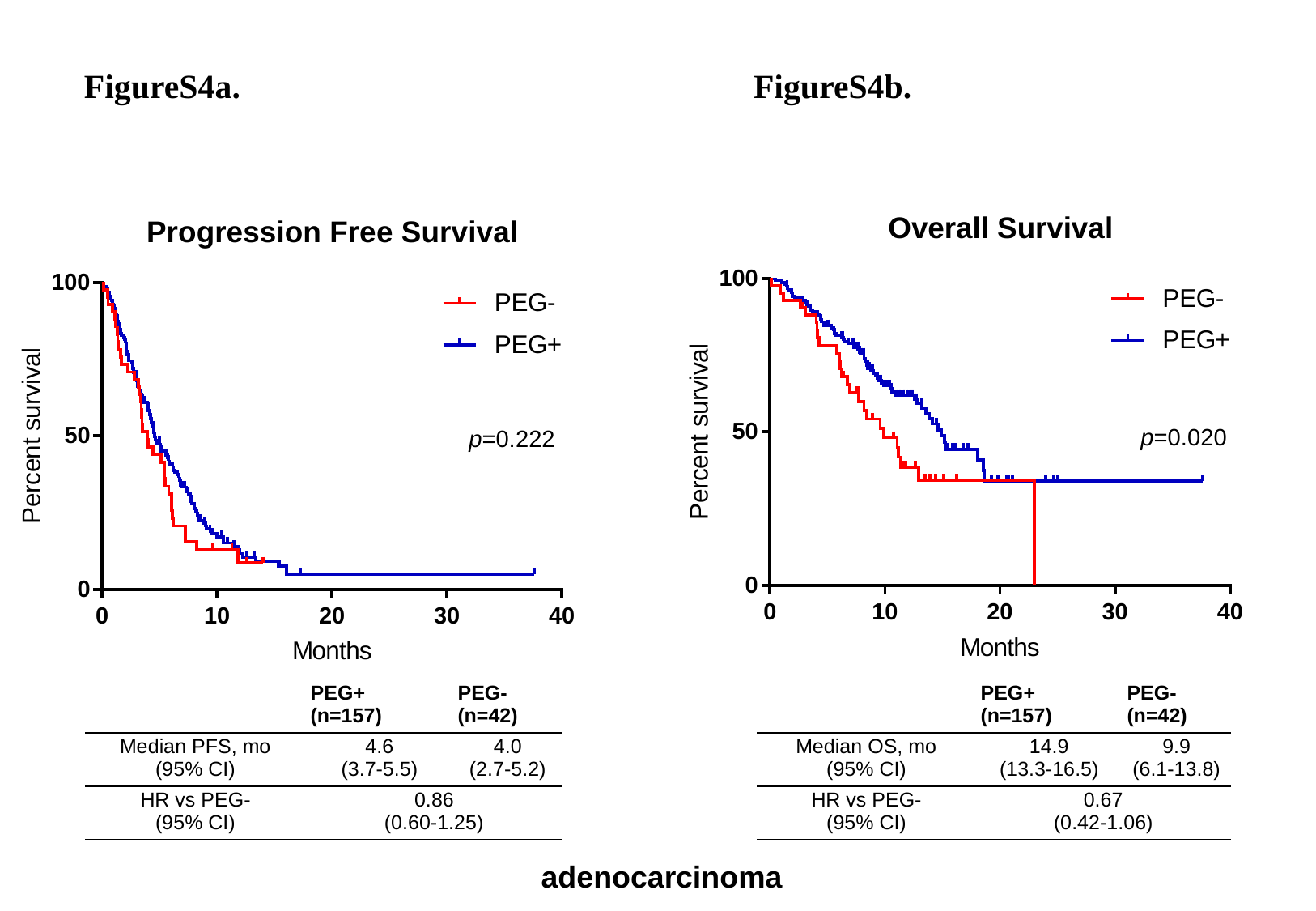

FigureS4a.
FigureS4b.
p=0.020
p=0.222
| | PEG+ (n=157) | PEG- (n=42) |
| --- | --- | --- |
| Median OS, mo (95% CI) | 14.9 (13.3-16.5) | 9.9 (6.1-13.8) |
| HR vs PEG- (95% CI) | 0.67 (0.42-1.06) | |
| | PEG+ (n=157) | PEG- (n=42) |
| --- | --- | --- |
| Median PFS, mo (95% CI) | 4.6 (3.7-5.5) | 4.0 (2.7-5.2) |
| HR vs PEG- (95% CI) | 0.86 (0.60-1.25) | |
adenocarcinoma

## Slide 5
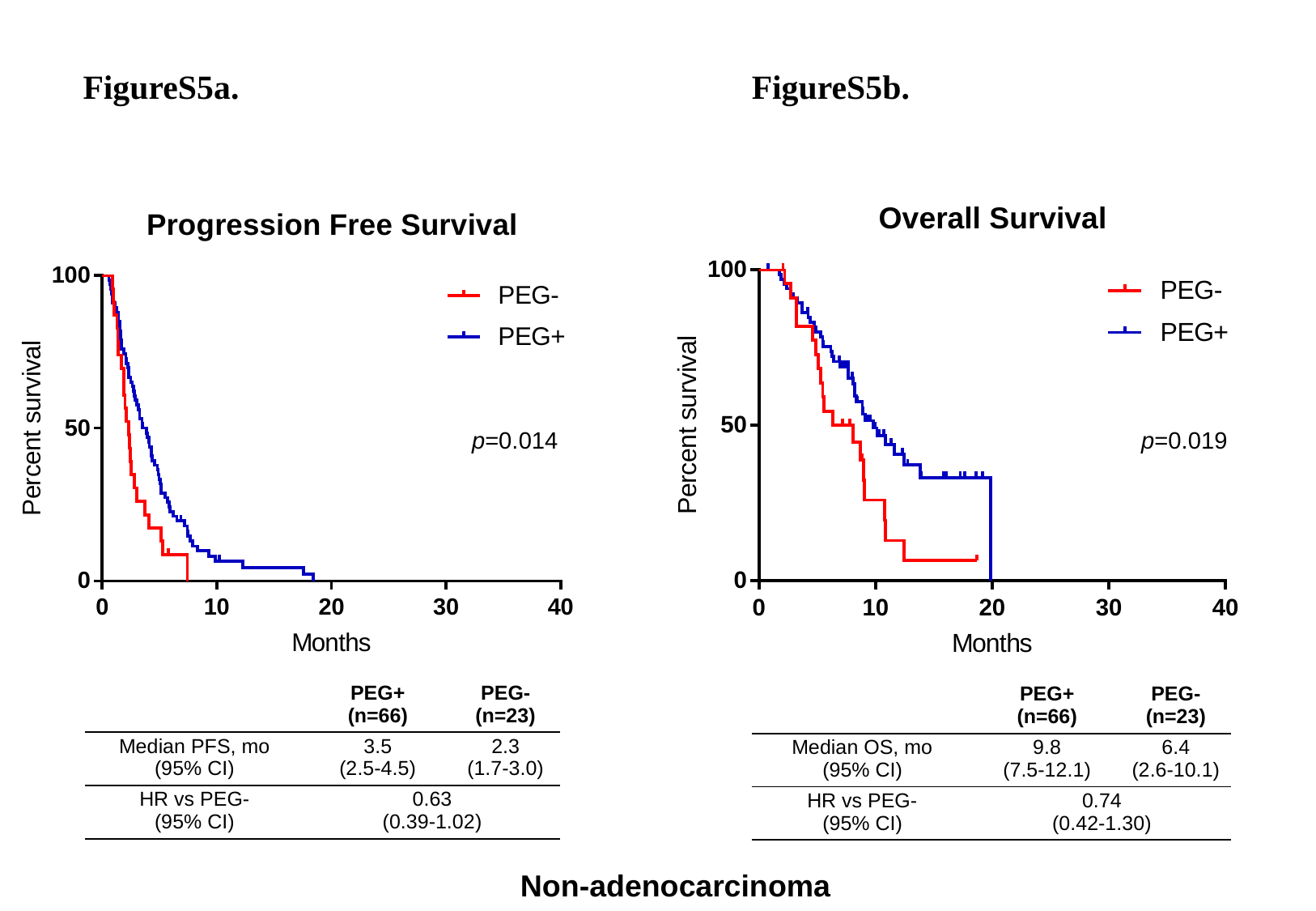

FigureS5b.
FigureS5a.
p=0.019
p=0.014
| | PEG+ (n=66) | PEG- (n=23) |
| --- | --- | --- |
| Median PFS, mo (95% CI) | 3.5 (2.5-4.5) | 2.3 (1.7-3.0) |
| HR vs PEG- (95% CI) | 0.63 (0.39-1.02) | |
| | PEG+ (n=66) | PEG- (n=23) |
| --- | --- | --- |
| Median OS, mo (95% CI) | 9.8 (7.5-12.1) | 6.4 (2.6-10.1) |
| HR vs PEG- (95% CI) | 0.74 (0.42-1.30) | |
Non-adenocarcinoma
