# Supplementary material for: Prophylactic pegfilgrastim reduces febrile neutropenia in ramucirumab plus docetaxel after chemoimmunotherapy in advanced NSCLC: post hoc analysis from NEJ051
Source: Sci Rep. 2024 Feb 15;14:3816. doi: 10.1038/s41598-024-54166-x (PMC10869351; doi:10.1038/s41598-024-54166-x)
Supplement: Supplementary file 2 — Supplementary Tables. [file 41598_2024_54166_MOESM2_ESM.docx]

**Table S1. The list of NEJ051 study’s participating institutions***

| 1 | Hokkaido University Hospital |
| --- | --- |
| 2 | Hakodate Goryoukaku Hospital |
| 3 | Asahikawa Medical University Hospital |
| 4 | KKR Sapporo Medical Centre |
| 5 | Hirosaki University Hospital |
| 6 | Iwate Medical University Hospital |
| 7 | Sendai Kousei Hospital |
| 8 | Tohoku University Hospital |
| 9 | Miyagi Cancer Center |
| 10 | Omagari Kosei Medical Center |
| 11 | Yamagata Prefectural Central Hospital |
| 12 | Fukushima Medical University Hospital |
| 13 | JA Mito Kyodo Hospital |
| 14 | Tsukuba University Hospital |
| 15 | Tsukuba Medical Center Hospital |
| 16 | Ibaraki Prefectural Central Hospital |
| 17 | Saiseikai Utsunomiya Hospital |
| 18 | Jichi Medical University Hospital |
| 19 | Local independent administrative corporation, Tochigi Prefectural Cancer Center |
| 20 | Dokkyo Medical University Hospital |
| 21 | Sano Kosei General Hospital |
| 22 | Gunma University Hospital |
| 23 | Gunma Prefectural Cancer Center |
| 24 | Saitama Red Cross Hospital |
| 25 | Saitama Cancer Center |
| 26 | Saitama Medical University International Medical Center |
| 27 | Jichi Medical University Saitama Medical Center |
| 28 | Saitama Medical University Hospital |
| 29 | Nishisaitama Chuo National Hospital |
| 30 | Juntendo University Graduate School of Medicine |
| 31 | The Cancer Institute Hospital of Japanese Foundation for Cancer Research |
| 32 | Nippon Medical School Hospital |
| 33 | Kyorin University Hospital |
| 34 | National Disaster Medical Center |
| 35 | Teikyo University Hospital |
| 36 | Toho University Medical Center Omori Hospital |
| 37 | Kitasato University Hospital |
| 38 | St. Marianna Medical University Hospital |
| 39 | Niigata Cancer Center Hospital |
| 40 | Niigata University Medical & Dental Hospital |
| 41 | Takaoka Koseiren Hospital |
| 42 | University of Fukui Hospital |
| 43 | Shinshu University |
| 44 | Saku General Hospital Saku Medical Center |
| 45 | Shizuoka Cancer Center |
| 46 | Matsusaka Municipal Hospital |
| 47 | Osaka International Cancer Institute |
| 48 | Kansai Medical University Hospital |
| 49 | NHO Kinki-Chuo Chest Medical Center |
| 50 | Kobe City Medical Center General Hospital |
| 51 | Kobe Minimally Invasive Cancer Center |
| 52 | Wakayama Medical University Hospital |
| 53 | Shimane University Hospital |
| 54 | NHO Yamaguchi Ube Medical Center |
| 55 | National Hospital Organization Iwakuni Clinical Center |
| 56 | National Hospital Organization Shikoku Cancer Center |
| 57 | Ehime University Hospital |
| 58 | JCHO Kyushu Hospital |
| 59 | Kurume University Hospital |
| 60 | Iizuka Hospital |
| 61 | Kitakyushu Municipal Medical Center |
| 62 | Nagasaki University Hospital |

*Arranged from north to south in order of the location

**Table S2.** **Efficacy of ramucirumab plus docetaxel according to PEG during the first cycle**

| **Response** | **Total**  n=288 | **PEG** | | |
| --- | --- | --- | --- | --- |
|  |  | **Yes**  n=223 | **No**  n=65 | ***p*-value** |
| CR | 1 | 1 | 0 | - |
| PR | 82 | 69 | 13 | - |
| SD | 118 | 91 | 27 | - |
| PD | 73 | 50 | 23 | - |
| NE | 14 | 12 | 2 | - |
| ORR, %  (95% CI) | 28.8  (23.7-34.4) | 31.4  (25.6-37.8) | 20.0  (11.9-31.4) | 0.087 |
| DCR, %  (95% CI) | 69.8  (64.1-75.0) | 72.2  (66.0-77.7) | 61.5  (49.4-72.4) | 0.124 |

Abbreviations: PEG, pegfilgrastim; CR, complete response; PR, partial response; SD, stable response; PD, progressive disease; NE, not evaluable; ORR, objective response rate; DCR, disease control rate; CI, confidence interval.
