# Supplementary material for: Prophylactic pegfilgrastim reduces febrile neutropenia in ramucirumab plus docetaxel after chemoimmunotherapy in advanced NSCLC: post hoc analysis from NEJ051
Source: Sci Rep. 2024 Feb 15;14:3816. doi: 10.1038/s41598-024-54166-x (PMC10869351; doi:10.1038/s41598-024-54166-x)
Supplement: Supplementary file 3 — Supplementary Information 3. [file 41598_2024_54166_MOESM3_ESM.docx]

**Supplemental figures**

**Figure S1:**

Kaplan–Meier curves showing progression free survival (S1a) and overall survival (S1b) classified according to whether prophylactic pegfilgrastim (PEG) was administered after the first cycle of ramucirumab plus docetaxel or not. Median progression free survival was 4.4 months (95% CI, 3.9-4.8) in the PEG prophylaxis group and 3.4 months (95% CI, 2.7-4.0) in the control group (*p*=0.022). Median overall survival was 13.8 months (95% CI, 11.8-15.8) in the PEG prophylaxis group and 8.7 months (95% CI, 7.2-10.1) in the control group (*p*=0.001).

**Figures S2 and S3:**

Kaplan–Meier curves showing progression-free survival (S2a) and overall survival (S2b), classified according to the presence or absence of prophylactic pegfilgrastim in ramucirumab plus docetaxel treatment. The upper row shows the analysis for adenocarcinoma (S3a) while the lower row shows that for non-adenocarcinoma (S3b).

**Figures S4 and S5:**

Kaplan–Meier curves showing progression-free survival (S4a) and overall survival (S4b), classified according to the presence or absence of prophylactic pegfilgrastim from the first cycle of ramucirumab plus docetaxel. The upper row shows the analysis for adenocarcinoma (S5a) the lower row shows that for non-adenocarcinoma (S5b).
